# Does medical disparity exist while treating severe mental illness patients with acute appendicitis in emergency departments? A real-world database study

**DOI:** 10.1186/s12888-022-04141-5

**Published:** 2022-07-21

**Authors:** Shang-Kai Hung, Hao-Wei Kou, Kai-Hsiang Wu, Shou-Yen Chen, Chih-Huang Li, Chao-Wei Lee, Yu-Yung Hung, Shi-Ying Gao, Po-Han Wu, Chiao-Hsuan Hsieh, Chung-Hsien Chaou

**Affiliations:** 1 Department of Emergency Medicine, Linkou Chang Gung Memorial Hospital, Taoyuan City, 333 Taiwan; 2 Department of Surgery, Division of General Surgery, Linkou Chang Gung Memorial Hospital, Taoyuan City, 333 Taiwan; 3 Department of Emergency Medicine, Chiayi Chang Gung Memorial Hospital, Chiayi County, 613 Taiwan; 4 Department of Nursing, Chang Gung University of Science and Technology, Chiayi Campus, Chiayi County, 613 Taiwan; 5 Graduate Institute of Clinical Medical Sciences, Division of Medical Education, College of Medicine, Chang Gung University, Taoyuan City, 333 Taiwan; 6 Graduate Institute of Clinical Medical Sciences, Chang Gung University, Taoyuan City, 333 Taiwan; 7 College of Medicine, Chang Gung University, Guishan, Taoyuan City, 333 Taiwan; 8 Department of Psychiatry, Kaohsiung Veterans General Hospital, Kaohsiung City, 813 Taiwan; 9 Chang-Gung Medical Education Research Centre, Chang-Gung Memorial Hospital, No. 5, Fusing St., Guei-shan Township, Taoyuan City, 333 Taiwan

**Keywords:** Severe mental illness, Acute appendicitis, Health disparity, Emergency department

## Abstract

**Background:**

Patients with severe mental illness (SMI) have a shorter life expectancy and have been considered by the World Health Organization (WHO) as a vulnerable group. As the causes for this mortality gap are complex, clarification regarding the contributing factors is crucial to improving the health care of SMI patients. Acute appendicitis is one of the most common indications for emergency surgery worldwide. A higher perforation rate has been found among psychiatric patients. This study aims to evaluate the differences in appendiceal perforation rate, emergency department (ED) management, in-hospital outcomes, and in-hospital expenditure among acute appendicitis patients with or without SMI via the use of a multi-centre database.

**Methods:**

Relying on Chang Gung Research Database (CGRD) for data, we selectively used its data from January 1st, 2007 to December 31st, 2017. The diagnoses of acute appendicitis and SMI were confirmed by combining ICD codes with relevant medical records. A non-SMI patient group was matched at the ratio of 1:3 by using the Greedy algorithm. The outcomes were appendiceal perforation rate, ED treatment, in-hospital outcome, and in-hospital expenditure.

**Results:**

A total of 25,766 patients from seven hospitals over a span of 11 years were recruited; among them, 11,513 were excluded by criteria, with 14,253 patients left for analysis. SMI group was older (50.5 vs. 44.4 years, *p* < 0.01) and had a higher percentage of females (56.5 vs. 44.4%, *p* = 0.01) and Charlson Comorbidity Index. An analysis of the matched group has revealed that the SMI group has a higher unscheduled 72-hour revisit to ED (17.9 vs. 10.4%, p = 0.01). There was no significant difference in appendiceal perforation rate, ED treatment, in-hospital outcome, and in-hospital expenditure.

**Conclusions:**

Our study demonstrated no obvious differences in appendiceal perforation rate, ED management, in-hospital outcomes, and in-hospital expenditure among SMI and non-SMI patients with acute appendicitis. A higher unscheduled 72-hour ED revisit rate prior to the diagnosis of acute appendicitis in the SMI group was found. ED health providers need to be cautious when it comes to SMI patients with vague symptoms or unspecified abdominal complaints.

**Supplementary Information:**

The online version contains supplementary material available at 10.1186/s12888-022-04141-5.

## Background

Patients with severe mental illness (SMI) have a shorter life expectancy and have been considered by the World Health Organization (WHO) as a vulnerable group [1, 2]. This mortality gap is estimated to shorten by 13 to 30 years among SMI patients, and 60% of this excess mortality is attributed to physical illness [3, 4]. Even in countries with high-quality health care systems, the gap still exists and has, in fact, widened in recent decades [5]. The causes for this mortality gap are complex and multifactorial. The patient-level factors include higher rates of suicide, accidental and violent death, poorer self-care, and higher occurrence of physical diseases; the socioeconomic-level factors include lower economic support, social stigma, and structural discrimination; and the health service-level factors include the iatrogenic adverse effect of some psychiatric medications, inequalities in physical comorbidity, and poorer access to emergent healthcare resources [6–8]. To clarify the contributing factors, it is crucial to improve the health care of SMI patients and further narrow down the disparity gap.

Acute appendicitis is one of the most common indications for emergency surgery worldwide, having an estimated 7–8% lifetime risk [9]. Although the specific pathophysiology of acute appendicitis is still unknown, inflammation secondary to direct luminal obstruction caused by a fecalith, lymphoid hyperplasia, or impacted stool is considered a major trigger. Given the variable presentations of acute appendicitis, the diagnostic values of clinical symptoms and signs are low. The most commonly used image modality in adult patients is the Computed Tomography (CT) scan. Surgical intervention is the cornerstone of treatment, and delay in management is associated with increased perforation rates [10]. Due to the high incidence of acute appendicitis and the effectiveness of surgical interventions, appendectomy is considered the essential surgical service for basic human rights and is an indicator to evaluate health disparities [11, 12].

Earlier literature found a higher appendiceal perforation rate among vulnerable groups and considered the phenomenon as a disparity [13, 14]. It is unclear whether the disparity still exists within the SMI patient population and what potential factors contribute to this phenomenon. This study aims to evaluate the differences in appendiceal perforation rate, emergency department (ED) management, in-hospital outcomes, and in-hospital expenditure experienced by acute appendicitis patients with or without SMI via the use of a multi-centre database.

## Methods

### Data source

We obtained our data from the Chang Gung Research Database (CGRD), the largest multi-institutional electronic medical records (EMR) collection in Taiwan. CGRD collects data from seven Chang Gung Memorial Hospitals (CGMH), including two tertiary medical centres, two regional hospitals, and three district hospitals. CGMH has a 10,050 beds capacity and admits more than a million patients each year [15]. According to government statistics, there were over 500,000 ED visits to CGMH in 2015 and CGMH annually receives 11.5% of the National Health Insurance budget in Taiwan [16]. The basic architecture for CGRD includes clinical, epidemiological, laboratory, nursing, and disease categories, and cancer registry data for inpatient, outpatient, and emergency patients. The CGRD has been collecting de-identified data on the current and previous health conditions of patients by using the International Classification of Diseases, Ninth Revision, Clinical Modification (ICD-9-CM) codes before 2016 and using ICD-10-CM codes afterwards [17].

### Study population

The study subjects were selected based on the CGRD data from January 1st, 2007 to December 31st, 2017. The diagnoses of acute appendicitis were confirmed by the ICD codes in discharge medical records combined with the national health insurance declarations data. Relevant diagnostic ICD codes identified the diagnoses of SMI for schizophrenia, bipolar disorder, or major depressive disorder by the psychiatrist at least once before the diagnosis of acute appendicitis. The exclusion criteria were below 18 years of age, transferred from other health facilities and incomplete medical records. The relevant ICD codes for acute appendicitis and SMI are listed in Table 1.

**Table 1:** ICD codes for inclusion criteria

	ICD-9-CM code	ICD-10-CM code
**Acute Appendicitis**		
Perforation	540.0; 540.1	K352; K353
Non-perforation	541; 542	K35; K3580; K3589; K36; K37
**SMI**^**a**^		
Depression	296.20–26; 296.30–36, 296.82, 298.0	F32.0–5; F32.9; F33.0–4; F33.9; F32.8
Schizophrenia	295; 297; 298.3–4; 298.9	F20.0–3; F20.5; F20.8–9; F25.0–1; F25.8–9 F22–24; F28–29
Bipolar disorder	296.00–16; 296.40–81; 296.89–99; 298.1	F30.1–4; F30.8–9; F31.0–9; F28

^a^*SMI* Severe mental illness

### Study outcomes and covariates

Patients in the CGRD who were hospitalized through ED under the diagnosis of acute appendicitis were identified. The outcomes were appendiceal perforation rate, ED treatment, in-hospital outcome, and in-hospital expenditure. ED treatment consisted of analgesics usage, opioid analgesics, and non-opioid analgesics usage, and time from triage to first order, time from triage to first antibiotics administration, time from triage to receive CT scan, and time from triage to surgical consultation. The in-hospital outcome consisted of admission day, ICU admission, and in-hospital mortality. Appendiceal perforation has been identified as ICD-9-CM codes 540.0 and 540.1 or ICD-10-CM codes K35.2 and K35.3. In-hospital mortality has been defined as mortality during the hospitalization by any etiology. The in-hospital expenditure was extracted from national health insurance declarations data. The covariates include demographic data, triage data, vital signs during ED stay, laboratory data, Charlson Comorbidity Index (CCI), operative methods, and unscheduled 72-hour revisit before the diagnosis of acute appendicitis. CCI is calculated based on the sum of weighted diagnoses, including several comorbidities [18]. We determined the CCI score of patients based on there being two or more OPD visits with the same diagnosis in 1 year. CCI scores were categorized into three grades: mild, with CCI scores of 1–2; moderate, with CCI scores of 3–4; and severe, with CCI scores ≧ 5 [19]. The covariates include acute psychiatric ward admission in the past 1 year, length of stay in acute psychiatric ward in the past 1 year, medications include antipsychotics, antidepressant, benzodiazepine and mood stabilizer were identified in SMI group.

### Statistical analysis

The continuous variables are presented as mean (SD), and the categorical variables are presented as count (%). Given the large gap in the numbers of patients between the two groups, we utilized a matched case-control study design. A non-SMI patient group was matched at a ratio of 1:3 by using the Greedy algorithm [20]. The variables included in the matching process were age (±5 years) and gender. We compared the continuous variables between the two independent groups by using the Student’s t-test and compared the categorical variables between the two independent groups by using the Chi-square test. The statistical analysis is done using SAS version 9.4. A *p*-value of less than 0.05 is considered statistically significant. The work has been reported in line with the Strengthening The Reporting of Observational Studies in Epidemiology (STROBE) criteria [21].

### Ethical approval

This study was approved by the Chang Gung Medical Foundation Institutional Review Board (IRB: 202001785B0), waiving the need for obtaining the informed consent of the study participants.

## Results

### Patient characteristics

**Fig. 1:**
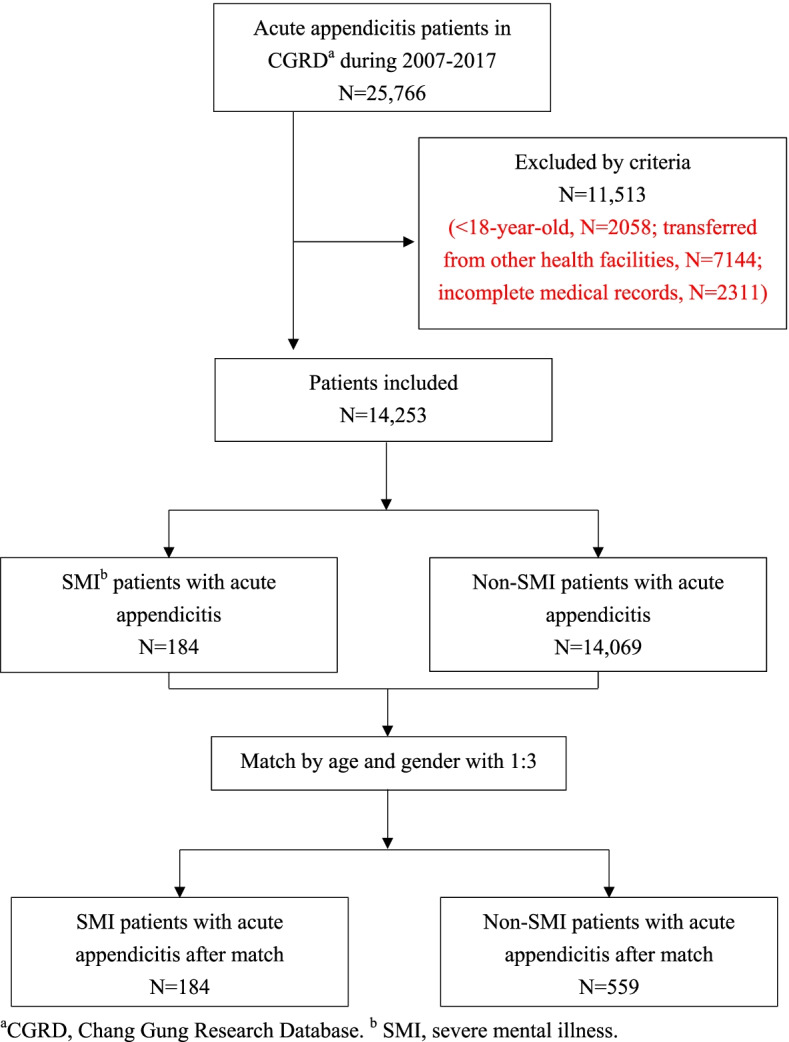
Flow chart of participants in cohort study. **a** CGRD, Chang Gung Research Database. **b** SMI, severe mental illness

A total of 25,766 patients from seven hospitals over a span of 11 years were recruited; of these, 11,513 were excluded by criteria, with 14,253 patients left for analysis. The recruitment flowchart is summarized in Fig. 1. The mean age of the participants is 44.5 ± 17.5 (years). Of all the participants, 47.0% were female. In the SMI group, 64.1% of the patients had depression, 27.7% had schizophrenia, and 8.2% had bipolar disorder. When the patient demographics and medical history of both the groups were compared, we found the SMI group to be older (50.5 vs. 44.4 years, *p* < 0.01) and have a higher percentage of females (56.5 vs. 44.4%, *p* = 0.01). Comorbidities including cardiovascular disease (21.2 vs. 7.1%, *p* < 0.01), cerebrovascular disease (22.8 vs. 8.6%, p < 0.01), pulmonary disease (33.7 vs. 14.6%, *p* < 0.01), liver disease (43.5 vs. 19.1%, p < 0.01), diabetes mellitus (32.6 vs. 11.7%, p < 0.01), renal disease (16.9 vs. 7.4%, *p* < 0.01), and malignancy (13.0 vs. 8.3%, *p* = 0.03) had a higher prevalence in the SMI group. SMI group also had a higher percentage of patients placed in moderate to severe CCI stages (*p* < 0.01). After matching with age and gender, comorbidities including cardiovascular disease (21.2 vs. 9.3%, *p* < 0.01), cerebrovascular disease (22.8 vs. 11.1%, *p* = 0.01), pulmonary disease (33.7 vs. 16.8%, *p* < 0.01), liver disease (43.5 vs. 23.3%, p < 0.01), diabetes mellitus (32.6 vs. 14.3%, p < 0.01), and renal disease (16.9 vs. 8.4%, p < 0.01) still showed a higher prevalence in the SMI group with a higher percentage of patients placed in moderate to severe CCI stages (p < 0.01). The above results were summarized in Table 2.

**Table 2:** Patient characteristics for overall, non-matched and matched groups

Variable	Overall (*N* = 14,253)	Non-match			Matched by age and gender (1:3)		
		SMI^a^ (*N* = 184)	Non-SMI (*N* = 14,069)	*p* value	SMI (*N* = 184)	Non-SMI (*N* = 559)	*p* value
Age (year)	44.5 ± 17.5	50.5 ± 16.1	44.4 ± 17.5	< 0.01*	50.5 ± 16.1	50.5 ± 16.0	0.96
Female	6696 (47.0)	104 (56.5)	6592 (46.9)	0.01*	104 (56.5)	318 (56.9)	0.99
**Categories of SMI**							
Depression	118 (64.1)	118 (64.1)	–	–	118 (64.1)	–	–
Schizophrenia	51 (27.7)	51 (27.7)	–	–	51 (27.7)	–	–
Bipolar disorder	15 (8.2)	15 (8.2)	–	–	15 (8.2)	–	–
**Comorbidities**							
Cardiovascular disease	1040 (7.3)	39 (21.2)	1001 (7.1)	< 0.01*	39 (21.2)	52 (9.3)	< 0.01*
Cerebrovascular disease	1246 (8.7)	42 (22.8)	1204 (8.6)	< 0.01*	42 (22.8)	62 (11.1)	< 0.01*
Pulmonary disease	2118 (14.9)	62 (33.7)	2056 (14.6)	< 0.01*	62 (33.7)	94 (16.8)	< 0.01*
Liver disease	2766 (19.4)	80 (43.5)	2686 (19.1)	< 0.01*	80 (43.9)	130 (23.3)	< 0.01*
Diabetes mellitus	1700 (11.9)	60 (32.6)	1640 (11.7)	< 0.01*	60 (32.6)	80 (14.3)	< 0.01*
Renal disease	1070 (7.5)	31 (16.9)	1039 (7.4)	< 0.01*	31 (16.9)	47 (8.4)	< 0.01*
Malignancy	1195 (8.4)	24 (13.0)	1171 (8.3)	0.03*	24 (13.0)	53 (9.5)	0.22
AIDS^b^	13 (0.1)	1 (0.5)	12 (0.1)	0.16	1 (0.5)	0 (0)	0.25
CCI^c^ 1–2	10,432 (73.2)	90 (48.9)	10,342 (73.5)	< 0.01*	90 (48.9)	369 (66.0)	< 0.01*
CCI 3–4	225 7(15.8)	50 (27.2)	2207 (15.7)	< 0.01*	50 (27.2)	111 (19.9)	< 0.01*
CCI ≧5	1564 (11.0)	44 (23.9)	1520 (10.8)	< 0.01*	44 (23.9)	79 (14.1)	< 0.01*

Count data are expressed as number (percentage) and continuous values are expressed as mean ± SD

^a^*SMI* Severe mental illness. ^b^*AIDS* Acquired immune deficiency syndrome. ^c^Charlson Comorbidity Index

*p < 0.05

Further analysis of the matched group revealed SMI group to have a higher triage pulse rate (94.1 vs. 90.0 times/min, *p* = 0.01), lower sodium level (136.8 vs. 137.7 mEq/L, *p* = 0.03) and higher unscheduled 72-hour revisit to ED (17.9 vs. 10.4%, p = 0.01). ED management, including opioid and non-opioid analgesics prescription, time to first order, time to the first antibiotics treatment, time to receive CT scan, and time to surgical consultation, for the two groups had no significant difference. In-hospital expenditure and in-hospital outcomes, including admission day, ICU admission rate, and appendiceal perforation rate, for the two groups also had no significant difference. The above results were summarized in Table 3.

**Table 3:** Vital signs, laboratory test, operative methods, ED treatment and hospital outcomes for matched group

Variable	SMI^**a**^ (*N* = 184)	Non-SMI (*N* = 559)	***p*** value
**Triage**^j^			0.91
1	1 (0.5)	3 (0.5)	
2	24 (13.0)	66 (11.8)	
3	153 (83.2)	464 (83.0)	
4	6 (3.3)	25 (4.5)	
5	0 (0)	1 (0.2)	
**Vital signs in triage**			
GCS^b^	14.6 ± 0.5	15.0 ± 0.3	0.56
Pulse Rate (times/min)	94.1 ± 19.7	90.0 ± 17.4	0.01*
Systolic blood pressure (mmHg)	136.4 ± 27.5	136.2 ± 24.6	0.92
Respiratory rate (times/min)	18.6 ± 1.5	18.5 ± 1.5	0.57
Body temperature (°C)	37.0 ± 1.1	37.0 ± 0.9	0.66
**Laboratory test**			
White cell count (1000/uL)	13.4 ± 5.0	13.4 ± 4.8	0.93
Platelet (1000/uL)	216.7 ± 66.3	224.3 ± 69.7	0.21
Prothrombin time (sec)	4.9 ± 5.0	4.8 ± 4.9	0.84
APTT^c^ (sec)	30.8 ± 5.9	30.1 ± 3.1	0.48
BUN^d^ (mg/dL)	14.3 ± 11.0	14.9 ± 12.3	0.76
Creatinine (mg/dL)	1.1 ± 1.3	1.0 ± 1.0	0.59
Na (mEq/L)	136.8 ± 3.8	137.7 ± 3.1	0.03*
K (mEq/L)	3.8 ± 0.5	3.8 ± 0.4	0.51
ALT^e^ (U/L)	28.9 ± 18.6	26.1 ± 18.1	0.17
Sugar (mg/dL)	127.4 ± 45.8	126.0 ± 37.0	0.80
CRP^f^ (mg/L)	74.7 ± 101.3	67.4 ± 83.6	0.42
**Operative methods**			0.45
Laparotomy	60 (34.5)	209 (39.7)	
Laparoscopy	99 (56.9)	272 (51.6)	
Other	15 (8.6)	46 (8.7)	
**Appendiceal perforation**	67 (36.4)	191 (34.2)	0.64
**Unscheduled 72-hr ED revisit**	33 (17.9)	58 (10.4)	0.01*
**ED**^**g**^ **treatment**			
Analgesics	102 (55.4)	293(52.4)	0.53
Opioid analgesics	21 (20.6)	92 (31.4)	0.05
Non-opioid analgesics	81 (79.4)	201 (68.6)	0.05
Time to 1st order (min)	18.3 ± 11.6	18.1 ± 11.6	0.87
Time to 1st antibiotics (min)	161.0 ± 105.7	170.3 ± 136.4	0.37
Time to CT scan (min)	122.9 ± 137.6	126.6 ± 155.8	0.80
Time to surgical consultation (min)	161.9 ± 177.5	138.6 ± 146.4	0.13
**In-hospital outcome**			
Admission day	7.1 ± 6.0	6.3 ± 4.4	0.11
ICU^h^ admission	4 (2.2)	10 (1.8)	0.48
In-hospital mortality	0 (0)	0 (0)	–
In-hospital expenditure (TWD^i^)	51,018.1 ± 38,269.7	45,644.7 ± 35,165.2	0.08

Count data are expressed as number (percentage) and continuous values are expressed as mean ± SD

^a^*SMI* Severe mental illness. ^b^*GCS* Glasgow Coma Scale. ^c^*APTT* Activated partial thromboplastin time

^d^*BUN* Blood urea nitrogen. ^e^*ALT* Alanine aminotransferase. ^f^*CRP* C-reactive protein

^g^*ED* Emergency department. ^h^*ICU* Intensive care unit. ^i^*TWD* Taiwan dollar

^j^Five-Level Taiwan Triage and Acuity Scale

*p < 0.05

For SMI group, we further analyzed the psychiatric disease status, medication for SMI, ED treatment and in-hospital outcome according to SMI subgroup and there were no significant differences in psychiatric disease status, ED treatment and in-hospital outcomes. The results were summarized in Supplementary 1.

## Discussion

To the best of our knowledge, this study is the first to evaluate the emergency department management, in-hospital outcomes, and in-hospital expenditure experienced by acute appendicitis patients with or without severe mental illness. This real-world database study found no significant difference in appendiceal perforation rate between the SMI and non-SMI patients with acute appendicitis. ED management, in-hospital outcome, and in-hospital expenditure also showed no significant differences between these two groups.

Previous studies have found a higher appendiceal perforation rate among vulnerable groups, including elderly, racial minority, immigrant, poor socioeconomic, and insurance status populations, in comparison to the other groups [14, 22–24]. This correlation was also demonstrated in the case of psychiatric patients and considered to be an instance of medical disparity. Using the Taiwan national insurance health data of 1997 to 2001, Tsay et al. published an article in 2007 proving that SMI patients have a 2.83 times higher risk of appendiceal perforation than non-SMI patients with acute appendicitis [13, 25, 26]. However, we found no significant difference in appendiceal perforation rate between the SMI and non-SMI groups in this study. The evolution of medical treatment may explain this improvement. The increased utility of diagnostic modalities, including CT scans of acute appendicitis in the past few years, eliminates the medical gap in SMI patients [27, 28]. Besides, the national health insurance in Taiwan has shown a positive influence in narrowing down the financial gap and improving the outcomes provided to vulnerable groups [29]. The advantage of a near 100% population coverage rate with comprehensive expenditure coverage may alleviate the obstacles of medical accessibility faced by patients with SMI [30, 31].

Previous studies adopted analgesic prescription rate as an indicator to evaluate treatment disparity and proved the existence of a lower prescription rate among female and racial minorities during acute pain management [32–34]. We found no obvious differences in analgesic prescription rates between the SMI and non-SMI groups. However, a trend of lower opioid analgesics prescription rate was noticed among SMI patients. Several randomized trials have demonstrated that opioid analgesic is safe and efficient in treating acute appendicitis patients [35, 36]. Further, a previous study found a lower opioid prescription rate also among black paediatric children with acute appendicitis in the United States and considered this phenomenon as that of treatment inequity [37]. Although our study found no statistical significance among the two groups, the trend of lower opioid administration in SMI patients still needs further investigation. The waiting time for medical evaluation and treatment is also important to evaluate the potential disparities in ED. Previous studies have found that patients with mental illness experience a longer waiting time to see a physician in ED [38]. To understand the medical management in ED more comprehensively, we analysed time from triage to first order, time from triage to first antibiotics administration, time from triage to receiving CT scan, and time from triage to surgical consultation in ED. None of the above variables showed differences between the SMI and non-SMI groups.

Our study aligns with the prior research that proved SMI patients to have a higher prevalence of several physical comorbidities, including cardiovascular disease, cerebrovascular disease, pulmonary disease, liver disease, DM, and renal disease [39]. Although SMI patients with acute appendicitis have a higher prevalence of comorbidities than the general population, we found no obvious differences in admission day, ICU admission, in-hospital mortality, and in-hospital expenditure between the two groups. This may be because acute appendicitis is a relatively benign disease with extremely low postoperative major adverse effects and mortality rate. Even in patients with multiple comorbidities, the current management can treat effectively without excessive cost [40].

We found a higher rate of unscheduled 72-hour ED revisits prior to the diagnosis of acute appendicitis in the SMI group. This may be because of the following reasons. First, SMI patients have a higher emergency medical resource usage in the case of psychiatric or physical illness [41]. Second, the higher unscheduled 72-hour ED revisits rate may associate with misdiagnosis for prior ED visits. The possible risk factors include lower pain perception, poorer communication of SMI patients, and diagnostic overshadowing of health providers [42]. Diagnostic overshadowing, the misattribution of physical symptoms to mental illness, was proved to exist among ED health providers [43]. The superimposition of this stigmatizing attitude by the cognitive impairment and excess negative symptoms of SMI patients may make it difficult to have a timely diagnosis [44]. ED health providers have to be more cautious when it comes to SMI patients with vague symptoms or unspecified abdominal complaints, and multidisciplinary evaluation may benefit these vulnerable groups [45].

## Limitations

There are remaining limitations of this study. First, due to the nature of the database study, some crucial clinical details, including individual symptoms, mental status of SMI patients, physical examinations, and bedside ultrasonography results, were not included in the pre-designed data format. This hampered further analysis owing to the potential reasons of higher unscheduled 72-hr ED revisit, which may be a result of multi-factors. Second, although CGRD is one of the largest databases in Taiwan, we still missed the patients with SMI diagnoses or those who received appendectomies in other medical facilities. Third, there are many components of disparities in the medical field. Although we demonstrated the cardinal management and timeline in ED without there being any obvious difference among the SMI patients, further investigations on the overshadowing or stigmatizing attitudes of health providers are still needed.

## Conclusion

Our study demonstrated no obvious differences in appendiceal perforation rate, ED management, in-hospital outcomes, and in-hospital expenditure between the SMI and non-SMI patients with acute appendicitis. It found a higher unscheduled 72-hour ED revisit rate prior to the diagnosis of acute appendicitis in the SMI group. ED health providers need to be cautious of SMI patients with vague symptoms or unspecified abdominal complaints. There is a need for further investigation on the other components of medical disparities, including the overshadowing effect.

## Supplementary Information

**Additional file 1.**

**Additional file 2: Supplementary 1.** Psychiatric disease status and clinical outcomes for SMI subgroup.

## Data Availability

The datasets generated and/or analyzed during the current study are available from the corresponding author on reasonable request.
