# Supplementary material for: Does medical disparity exist while treating severe mental illness patients with acute appendicitis in emergency departments? A real-world database study
Source: BMC Psychiatry. 2022 Jul 21;22:488. doi: 10.1186/s12888-022-04141-5 (PMC9306199; doi:10.1186/s12888-022-04141-5)
Supplement: Supplementary file 2 — Additional file 2: Supplementary 1. Psychiatric disease status and clinical outcomes for SMI subgroup. [file 12888_2022_4141_MOESM2_ESM.docx]

Supplementary 1. Psychiatric disease status and clinical outcomes for SMI subgroup

| **Variable** | **Overall**  (N=184) | **Depression**  (N= 118) | **Schizophrenia**  (N= 51) | **Bipolar disorder**  (N= 15) | **p value** |
| --- | --- | --- | --- | --- | --- |
| **Psychiatric disease status** | | | | | |
| Acute psychiatric ward admission in the past one year | 6 (3.3) | 2 (1.7) | 4 (7.8) | 0 (0) | 0.0899 |
| LOS^a^ in acute psychiatric ward in the past one year | 29.17 (25.8) | 28.5 (7.8) | 29.5 (33.0) | - | 0.9700 |
| Somatization disorder | 0 (0) | 0 (0) | 0 (0) | 0 (0) | - |
| **Medication for SMI^b^** | | | | | |
| Antipsychotic | 82 (44.6) | 44 (37.3) | 29 (56.9) | 9 (60.0) | 0.0288* |
| Antidepressant | 88 (47.8) | 69 (58.5) | 14 (27.5) | 5 (33.3) | 0.0005* |
| Benzodiazepine | 122 (66.3) | 80 (67.8) | 34(66.7) | 8 (53.3) | 0.5352 |
| Mood stabilizer | 19 (10.3) | 8 (6.8) | 6 (11.8) | 5 (33.3) | 0.0058* |
| **ED^c^ treatment** | | | | | |
| Appendiceal perforation | 67 (36.4) | 36 (30.5) | 25 (49.0) | 6 (40.0) | 0.0685 |
| Unscheduled 72-hr ED revisit | 33 (17.9) | 23 (19.5) | 8 (15.7) | 2 (13.3) | 0.7463 |
| Analgesics | 102 (55.4) | 65 (55.1) | 26 (51.0) | 11 (73.3) | 0.3072 |
| Opioid analgesics | 21 (20.6) | 16 (13.6) | 2 (3.9) | 3 (20.0) | 0.2121 |
| Non-opioid analgesics | 81 (79.4) | 49 (41.5) | 24 (47.1) | 8 (53.3) | 0.2121 |
| Time to 1^st^ order (min) | 18.3±11.6 | 18.0±11.0 | 18.7±13.0 | 19.2±12.3 | 0.9130 |
| Time to 1^st^ antibiotics (min) | 161.0±105.7 | 170.3±112.0 | 146.5±92.5 | 131.3±86.9 | 0.2838 |
| Time to CT scan (min) | 123.0±137.6 | 111.3±113.2 | 130.6±135.1 | 183.0±257.0 | 0.1960 |
| Time to surgical consultation (min) | 161.9±177.5 | 160.3±178.6 | 147.8±110.1 | 213.7±296.1 | 0.4621 |
| **In-hospital outcome** | | | | | |
| Admission day | 7.1±6.0 | 7.1±6.1 | 7.7±6.3 | 5.4±3.3 | 0.4283 |
| ICU^d^ admission | 4 (2.2) | 0 (0) | 3 (5.9) | 1 (6.7) | 0.0967 |
| In-hospital mortality | 0 (0) | 0 (0) | 0 (0) | 0 (0) | - |
| In-hospital expenditure (TWD^e^) | 51018.1±38269.7 | 49810.1±34963.1 | 55207.6±47182.4 | 46275.9±29616.5 | 0.6215 |

Count data are expressed as number (percentage) and continuous values are expressed as mean ± SD.

^a^LOS, length of stay. ^b^SMI, severe mental illness.^c^ ED, emergency department.

^d^ ICU, intensive care unit. ^e^TWD, taiwan dollar. *p < 0.05
